# Neurodevelopmental disorders and microcephaly: how apoptosis, the cell cycle, tau and amyloid-β precursor protein APPly

**DOI:** 10.3389/fnmol.2023.1201723

**Published:** 2023-09-22

**Authors:** Deborah K. Sokol, Debomoy K. Lahiri

**Affiliations:** ^1^Section of Pediatrics, Department of Neurology, Indiana University School of Medicine, Indianapolis, IN, United States; ^2^Department of Psychiatry, Indiana University School of Medicine, Indianapolis, IN, United States; ^3^Department of Medical and Molecular Genetics, Indiana University School of Medicine, Indianapolis, IN, United States

**Keywords:** ASD, microcephaly, amyloid precursor protein, T21, Down’s syndrome, Alzheimer’s disease

## Abstract

Recent studies promote new interest in the intersectionality between autism spectrum disorder (ASD) and Alzheimer’s Disease. We have reported high levels of Amyloid-β Precursor Protein (APP) and secreted APP-alpha (sAPP a ) and low levels of amyloid-beta (Aβ) peptides 1–40 and 1–42 (Aβ40, Aβ42) in plasma and brain tissue from children with ASD. A higher incidence of microcephaly (head circumference less than the 3^rd^ percentile) associates with ASD compared to head size in individuals with typical development. The role of Aβ peptides as contributors to acquired microcephaly in ASD is proposed. Aβ may lead to microcephaly via disruption of neurogenesis, elongation of the G1/S cell cycle, and arrested cell cycle promoting apoptosis. As the APP gene exists on Chromosome 21, excess Aβ peptides occur in Trisomy 21-T21 (Down’s Syndrome). Microcephaly and some forms of ASD associate with T21, and therefore potential mechanisms underlying these associations will be examined in this review. Aβ peptides’ role in other neurodevelopmental disorders that feature ASD and acquired microcephaly are reviewed, including dup 15q11.2-q13, Angelman and Rett syndrome.

## Introduction

Over recent years there has been much interest in how the Amyloid-β Precursor Protein (APP) may contribute to autism spectrum disorder (ASD) and Fragile X Syndrome (FXS), particularly to the macrocephaly often seen in these disorders (Sokol and Lahiri, 2023). Particular molecular mechanisms suggest how APP may contribute to brain overgrowth in ASD and its single gene kin, Fragile X Syndrome (FXS) (Sokol et al., 2011; Bailey et al., 2013; Lahiri, et al., 2013; Westmark, 2019). Although macrocephaly is more typical of the disorder, a higher incidence of microcephaly (head circumference less than the 3^rd^ percentile) also associates with ASD compared to head size in individuals with typical development (Fombonne et al., 1999; Gillberg and de Souza, 2002; Cederlund, 2021). Aβ peptide, the APP fragment associated with plaques in Alzheimer’s disease (AD), may lead to microcephaly via disruption of neurogenesis, elongation of the G1/S cell cycle, and arrested cell cycle promoting apoptosis. As the APP gene exists on Chromosome 21, excess Aβ peptides occur in Trisomy 21-T21 (Down’s Syndrome). Microcephaly and some forms of ASD associate with T21, and therefore potential mechanisms underlying these associations will be examined in this review. Aβ peptides’ role in other neurodevelopmental disorders that feature ASD and acquired microcephaly are reviewed, including dup 15q11.2-q13, Angelman and Rett syndrome. It should be noted that these syndromes are relatively rare disorders compared to ASD without microcephaly and may not be typical of ASD in general.

### Intersection of Alzheimer’s disease and ASD

Recent studies and reviews have stimulated renewed interest in the intersectionality between ASD and Alzheimer’s Disease (AD; Nadeem et al., 2021; Hu et al., 2022; Rhodus et al., 2022). ASD is a developmental condition characterized by lifelong deficits in social–emotional reciprocity, in nonverbal communicative behaviors used for social interaction, and in developing, maintaining, and understand relationships, along with restrictive, repetitive patterns of behavior. ASD occurs in a ratio of 4 to 1 boys vs. girls. Macrocephaly (head circumference greater than the 98^th^ percentile) is seen more in boys and occurs early in development when symptoms become apparent (Sacco et al., 2015). In contrast to macrocephaly, reported to be as common as 37% (Courchesne et al., 2001), microcephaly is less common, i.e., 15.1% of a study involving 126 children with ASD (Fombonne et al., 1999). Microcephaly in ASD usually associates with epilepsy, intellectual deficiency and genetic abnormality (Hof et al., 1991; Guerin et al., 1996; Fombonne et al., 1999; Cederlund, 2021). For example, acquired microcephaly with ASD is seen in syndromes associated with transcription deficiencies: methyl-CpG binding protein 2-MeCP2 (Rett), Ubiquitin-protein ligase-UBE3A (Angelman), L-type voltage sensitive calcium channel-VSCC (Timothy) and CREB-binding protein-CBP (Ebert and Greenberg, 2013).

Alzheimer’s disease is a progressive neurological disorder, first evident as impaired memory, followed by loss of visuospatial skills, finally with impairment of speech and daily function. AD affects women more than men and is the most common form of dementia. A proteolytic metabolite of APP, Aβ peptide, is believed to be central to AD pathophysiology resulting in brain atrophy, as well as playing other important roles (Lahiri and Maloney, 2010). In AD there is accumulation of cerebral plaques composed of extracellular deposits of fibrillar Aβ peptide, along with intraneuronal neurofibrillary tangles (NFT) which are hyperphosphorylated cytoskeletal microtubule-associated protein Tau filaments. Plaques and NFT, together with impairment in neurogenesis contribute to the destruction of neurons in AD (Zeidan-Chulia et al., 2014).

Earlier, largely theoretical, reports suggested the anabolic brain processes in ASD would safeguard against developing dementia, associated with catabolic functions (Oberman and Pascual-Leone, 2014). Examples of anabolism found in ASD include the increase in number of neurons and excessive brain overgrowth (Courchesne et al., 2011; Marchetto et al., 2017), and an increase in secreted APP alpha (sAPPα) which is associated with upregulation of glutamatergic synapses and decline in GABAergic synapses (Ray et al., 2016; Rice et al., 2019; Tang, 2019). Examples of catabolism in dementia include the aforementioned accumulation of extracellular Aβ plaques and the intracellular tau NFTs. Contrary to the notion of “anabolic protection,” dementia turns out to be more prevalent in adults with ASD compared to the general population (Vivanti et al., 2021). In a case controlled nationwide study of adults aged 30–64 years enrolled in Medicaid, adults with ASD were 2.6 times more likely to show early-onset dementia (Vivanti et al., 2021). However, compared to individuals with intellectual deficiency, individuals with ASD and intellectual deficiency had a lower incidence of dementia (Vivanti et al., 2021). The survey had specific weaknesses acknowledged by its authors, such as the reported “dementia” could not be distinguished among AD, frontotemporal dementia, vascular dementia, or other dementia etiologies. Nevertheless, it presented an intriguing link between ASD and dementia that needs follow up in the population at large.

In a recent study, Rhodus et al., found several autistic behaviors such as social withdrawal, ritualistic behavior, resistance to change and avoidance of eye contact in a group of adults with AD and AD related dementias (Rhodus et al., 2022). These behaviors are not typically elicited on routine AD screens and addressing these unique behaviors was proposed to improve the quality of life for individuals with AD (Rhodus et al., 2022). Looking in the opposite direction, AD drugs have been used “off label” for ASD, including donepezil, galantamine, rivastigmine, memantine, and tacrine, resulting in several reports of improvement in core and co-morbid features of ASD (rev. Rossignol and Frye, 2014). The new monoclonal antibody drug therapies for AD, e.g., aducanumab, lecanemab, and donanemab, are designed to clear Aβ peptide, the chief component of amyloid plaques (van Dyck et al., 2023; Wessels et al., 2023; Wojtunik-Kulesza et al., 2023). This possibility bolsters our viewpoint that APP metabolites, including Aβ, may serve as therapeutic targets for conditions other than AD, such as T21 (Westmark et al., 2016). Aβ has been associated with the disruption of the cell cycle and mitosis with resultant apoptosis as seen in T21 (Granic et al., 2010; Judge et al., 2011). As microcephaly and ASD associates with T21, could Aβ contribute to microcephaly in other forms of ASD? This sets the stage for addressing how APP, particularly Aβ, may contribute to microcephaly in ASD.

### Amyloid-β precursor protein function and processing pathway

While extensively studied in AD, APP has roles beyond simply contributing to AD (Lahiri et al., 1998; Lahiri 1999; Bandyopadhyay et al., 2007). APP is produced in neurons, released from presynaptic terminals, and available at the dendritic synapse (Ishida et al., 1997; Weingarten et al., 2017). Constitutional APP (before processing) contributes to neurite growth (Mattson, 1997; Turner et al., 2003), gliogenesis (Kwak et al., 2010), synaptogenesis (Turner et al., 2003), neuroproliferation (Mattson, 1994), migration (Priller et al., 2006), suppression of cell adhesion (Chen and Bodles, 2007), and participates in translational pathways (Wang et al., 2004; Westmark and Malter, 2007).

APP is a large membrane spanning glycoprotein with a long extracellular N-terminus, a transmembrane region and an intracellular C-terminus, the APP intracellular domain (AICD; Kang et al., 1987). APP goes through translation in the endoplasmic reticulum and then undergoes post-translational modification (i.e., cleavage) in the Golgi complex before it travels to the cell membrane (Hefter and Draguhn, 2017). Once cleaved, APP metabolites are released in secreted forms. Post-translational proteolytic cleavage by β-site APP cleaving enzyme (BACE)1 via the amyloidogenic pathway forms secreted APPβ (sAPPβ). Subsequent cleavage of APP by 𝞬-secretase complex produces the AICD and Aβ peptides, which can aggregate into amyloid oligomers and fibrils. Amyloid oligomers are soluble and spread throughout the brain (Chen et al., 2017). Amyloid fibrils are larger, insoluble, and further assemble into AD plaques. However, it is the *nonamyloidogenic* pathway that is the predominant, constitutive path of APP proteolysis. This pathway is initiated by cleavage within the amino acid sequence preventing formation of the Aβ peptide, via a member of the α-secretase ADAM (A Disintegrin And Metalloprotease) 9,10,17 family, aka adamalysins, predominantly ADAM10. This produces secreted APP alpha (sAPPα), and cleavage by 𝜸-secretase produces AICD and the P3 peptide. Both sAPPα and P3 are neurotrophic and neuroprotective. Intracellular AICD translocates into the nucleus and activates gene transcription (Barucker et al., 2014). Post-translational phosphorylation of APP, particularly at the Threonine 668 (Thr^668^) site occurring in neurons, modulates the generation of Aβ (Zhang et al., 2019).

APP appears to regulate various components of neurogenesis, as suggested by its early expression during brain development (Nicolas and Hassan, 2014). For example, APP_695_, the form more prevalent in brain, has been found as early as mouse embryonic day (E) 6 within mesodermal cells of the primitive streak (Sarasa et al., 2000) and by E9.5 in the mouse neural tube, which coincides with the peak of neural differentiation and neurite outgrowth (Salbaum and Ruddle, 1994; Sarasa et al., 2000). sAPPα, the product of APP cleavage by α-secretase, functions similarly to growth factors. As such, sAPPα increases the *in vitro* proliferation of embryonic neural stem cells (NSCs) isolated from embryonic rat neocortex (Nicolas and Hassan, 2014). Murine proliferation of NSC (i.e., neurogenesis) naturally occurs E12-25, within the time frame of APP_695_ expression. In mouse embryonic neurons, APP undergoes fast axonal transport and accumulates in axonal growth cones and is released via an activity induced, calcium-mediated mechanism (Mattson and Furukawa, 1998). In mature mouse brain, APP appears in presynaptic terminals coincidentally with glutamate and may modulate postsynaptic response to glutamate; its delayed expression may result in natural cell death (Mattson and Furukawa, 1998).

In summary, the purported early arrival of APP in embryogenesis, the localization of APP at dendritic synapses, its role in cell adhesion, its interaction with post-translational pathways and in transcription suggests that APP metabolites may play a pivotal role in brain development and potentially, the development of ASD (Ray et al., 2016). The discovery of numerous inheritable and *de novo* gene variations localize to sites at the synapse, translation and post-translation and transcription in ASD (Ebert and Greenberg, 2013). We have proposed a role for APP and in particular nonamyloidogenic sAPPα, in brain overgrowth and macrocephaly in ASD (Sokol et al., 2019), and presently we look to the amyloidogenic pathway to explain microcephaly in ASD.

### Amyloid-β precursor protein in ASD

APP and its metabolites are dysregulated in ASD. We and others have reported high levels of APP and sAPP a (Sokol et al., 2006; Bailey et al., 2008; Ray et al., 2011, 2016; Frackowiak et al., 2013) and low levels of Aβ peptides 1–40 and 1–42 (Aβ40, Aβ42), in plasma and brain tissue from children with ASD. We interpreted our finding of low ASD brain Aβ40/Aβ42 as being consistent with the lack of fibrillar amyloid plaques in the brains of children with ASD. However, Al-Ayadhi et al. offered a different interpretation of their finding of low Aβ40/Aβ42 in the plasma of Saudi children with ASD (Al-Ayadhi et al., 2012). They speculated that the brain may in fact be a Aβ40, Aβ42 reservoir with decreased brain to blood efflux of Aβ peptide, explaining low Aβ40/Aβ42 in plasma, but, speculating high levels in brain. However, Wegiel et al. (2012) in fact, found no extracellular deposition of Aβ40/Aβ42 in the brains of older individuals with ASD, in nonfibrillar aggregations in the brains of older individuals with ASD. Including one with a genetic form of ASD (i.e., dup 15q11.2-q13). The APP-derived aggregates they reported were, instead P3, part of the nonamyloidogenic pathway. This finding contradicts Al-Ayadhi et al. (2012).

APP metabolites appear to be altered according to age in FXS, a single gene form of ASD. FXS is the most common form of inherited intellectual disability with a frequency of 1 in 2,500 births, as well as the leading known genetic cause of ASD (Hagerman, 2008). This X chromosome-linked disorder is clinically characterized by highly variable cognitive ability (overall IQ < 70), autistic-like behaviors, and macrocephaly. Seizures, although more common in ASD than in the general population, are not among the core features of ASD (Khundrakpam et al., 2017; Sokol et al., 2019). On a molecular level, Westmark and Malter discovered a FMRP regulated mGluR_5_/APP translational pathway linking APP to FXS (Westmark and Malter, 2007). mGluR_5_ may act as a master switch between the balance of catabolic and anabolic processes in nervous system development, maintained in part by regulating levels of APP metabolites, We have found increased plasma levels of APP and sAPP a in FXS vs. control subjects. In contrast, Westmark et al. (2011) found low levels of sAPP a and Aβ_42_ in adult FXS plasma and low levels of sAPP a but increased levels of Aβ_42_ in adult FXS brain suggesting that Aβ is sequestered in the adult FXS brain. To explain these age related findings, we (Westmark et al., 2016) proposed that a switch takes place in development: during youth, upregulated APP is processed by the α-secretase via the non-amyloidogenic pathway, releasing sAPPα. During aging, upregulated APP is processed by β-secretase releasing Aβ, following the amyloidogenic pathway. This parallels trends found in the population at large.

We have recently reported about how sAPPα levels contribute to increased white matter in ASD (Sokol et al., 2019). The purpose of the present paper is to review how the amyloidogenic pathway, particularly the proteolytic Aβ peptides and the AICD, may contribute to microcephaly which is associated with syndromic and some idiopathic forms of ASD. In this review we are most interested in the effect of Aβ40/Aβ42 on brain formation within conditions known to feature microcephaly and ASD such as dup 15q11.2-q13. After we describe normal brain formation, we use the T21 genetic condition as a prototype to explain how Aβ40/Aβ4 could influence microcephaly. T21 is a condition wherein microcephaly is a universal phenotype and APP is in abundance as APP localizes to chromosome 21 which is triplicated in T21.

### Normal brain growth

The size of the brain is determined by the number of constituent cells (neurons + glia) multiplied by the average size of each cell, both features linked by developmental pathways between neurogenesis and elements of the cell cycle (Ohnuma and Harris, 2003).

### Neurogenesis

Neurogenesis involves proliferation and differentiation of constituent brain cells (Ohnuma and Harris, 2003). Most of the neurons in the cerebral cortex are produced during mid-gestation, as proliferating progenitor neurons, that line the ventricle, i.e., the pseudostratified ventricular epithelium or ventricular zone (VZ). Mitosis takes place at the apical/ventricular surface. At first, new neurons proliferate symmetrically with each cell division producing two daughter cells; subsequently, approximately 28 cell cycles produce up to 400 neurons per founder cell in primate brains (Rakic, 1995). The neurons then migrate radially toward the pial cortical plate.(Rakic, 1995; Haydar et al., 2000) Neuronal nuclei travel up and down the pseudostratified epithelium, replicating their DNA at the cortical plate pial surface. Supporting our premise that APP may associate with processes underlying brain growth, APP was found in all VZ neurons in mice embryos in a study of neurogenesis (Lopez-Sanchez et al., 2005). Specifically, APP was found at the apical domain in cortical precursor cells during interphase. After interphase, APP re-localized peripherally around the metaphase plate, not far from the centrosome (Lopez-Sanchez et al., 2005).

Few neurons are generated after birth. Cerebral brain volume continues to grow without a change in the number of neurons due to the expansion of neuropil composed of axons, dendrites and glia (Gilmore and Walsh, 2013). The number of centrosomes, the cell’s microtubule organizing center, determine the number of neurites produced (Judge et al., 2011). Without centrosomes, the cells cannot divide properly, and mitosis fails (Judge et al., 2011). Further, centrosomes may play an important role in the development of microcephaly. Several of the genes discovered responsible for primary microcephaly associate with defective centrosome activity (Gilmore and Walsh, 2013). The largest increase in brain size occurs in the first year of life, despite the simultaneous pruning of connections. The final number of cortical neurons is determined by the progenitor founder cells present at the beginning of neurogenesis, by the proliferating progenitor neurons, and by cell death (Haydar et al., 2000). Increased processing of APP into Aβ and C-terminal fragments favor arrest of progression of these precursor founder cells through the cell cycle G1/S phase, resulting in gliogenesis instead of the formation of post mitotic neurons (Judge et al., 2011; Coronel et al., 2019).

### Cell cycle

The cell cycle impacts the size of the brain by directing growth via cell proliferation and cell growth (Herrup and Yang, 2007; Frade and Ovejero-Benito, 2015). The mammalian cell cycle is divided into four phases. Mitosis (M phase) occurs where cells divide and equally distribute their genetic material into 2 daughter cells. M phase is composed of Prophase when chromatin is compacted, nuclear membrane disappears and the mitotic spindle is formed; Metaphase when chromosomes line up at the equatorial plane; Anaphase when chromatids separate from the mitotic spindle and Telophase where the two sets of chromosomes arrive at the cell pole and two new daughter cell nuclei form (Herrup and Yang, 2007; Frade and Ovejero-Benito, 2015). Once the daughter cells are formed, they go through three subsequent phases: G1 where proteins needed for DNA/centrosome replication are made, S phase where nuclear DNA/centrosomes replicate and G2 where proteins required for cell division are made (Herrup and Yang, 2007; Frade and Ovejero-Benito, 2015). G1 is the period of growth before the cell divides. Brain cell size, a determinant of brain size, is controlled by cell growth during G1 until the brain is fully formed. G0 is a special form of G1, which involves differentiated cells unlikely to divide unless provoked. Fully differentiated, adult, post-mitotic neurons maintain their G0 quiescent state. Cell cycle progression (cell proliferation) and cell size regulation (cell growth) are yoked as DNA replication does not take place until cells reach a minimum size (Rosner et al., 2003). Progression through the cell cycle phases is regulated by checkpoints that make sure the cell has completed a phase before entering the subsequent one. If a defect is detected, these checkpoints block the cell cycle until the defect is repaired or by executing apoptosis if the defect is beyond repair (Herrup and Yang, 2007; Frade and Ovejero-Benito, 2015). The G1/S checkpoint ensures the cell is large enough to enter the DNA synthesis S phase. The G2/M checkpoint, right before mitosis, ensures DNA is not damaged to avoid transmission of mutation. The transitions between the phases are regulated by cyclins that bind to specific cyclin dependent kinases (Cdks), activating their kinase activity. Different signaling pathways at these checkpoints lead to the inhibition of the Cdk/cyclin units. The longer the cell cycle preventing completion, the fewer the number of cycles, and increase in cyclin dependent inhibitors all contribute to decreased number of neurons produced (Ohnuma and Harris, 2003).

Under pathological conditions such as AD, fully differentiated neurons emerge from G0 and attempt to re-enter the cell cycle (Vincent et al., 1997). This finding, together with that of upregulation of cell cycle regulatory proteins found in neurons of AD patients (discussed below) were thought to lead to neurodegeneration and apoptosis (Vincent et al., 1997; Herrup and Yang, 2007; Malik et al., 2008).

Components of the cell cycle significant to the discussion of microcephaly include cell cycle centrosome driven activity. More than half of the primary microcephaly genes encode proteins that involve the centrosome (Jayaraman et al., 2018). The centrosomes are composed of a mature mother centriole and a less mature daughter centriole that duplicate in G1/S phase and associate with microtubules that form the mitotic spindle. CDK5RAP2 is a gene that regulates CDK5, and when mutated, causes microcephaly (Gilmore and Walsh, 2013). CDK5RAP2 links two centrioles which go through G1/S to form a new centriole next to the parent centriole. The new centriole elongates in S phase and goes on to assist alignment of the mitotic spindle by which chromosomes are arranged in M phase (Gilmore and Walsh, 2013). Further, CDK5RAP2 potentiates tau protein phosphorylation (Miron et al., 2018), contributing to NFT, found to precede the neocortical deposition of Aβ in the entorhinal cortex from AD patients (Eftekharzadeh et al., 2018). Finally, as CDK5 increases phosphorylation of APP (Majd et al., 2019) its regulator, CDK5RAP2, may also promote APP phosphorylation and the generation of Aβ and C-terminal fragments.

### Amyloid-**β** precursor protein influence on cell cycle contributes to decreased neural progenitor cells

As the mitotic cell cycle produces new cells, the number of times the neural progenitor cells re-enter the cell cycle determines the size of the brain (Ohnuma and Harris, 2003). In normal cortical development, APP appears to play a role in cell cycle progression and mitosis (Lopez-Sanchez et al., 2005). As previously cited, APP exists in all VZ neurons where mitosis takes place in mouse embryos (Lopez-Sanchez et al., 2005). The duration of mitosis increased and the timing of entrance into G2 altered for the cortical precursor cells of APP-deficient mice. This suggested APP may influence the cell cycle via upregulation of cell cycle inhibitors and by supporting re-entry of cortical neurons into the mitotic cycle, favoring apoptosis (Lopez-Sanchez et al., 2005).

Aβ was shown to directly interfere with mitosis via disruption of the cytoskeleton leading to chromosome mis-segregation in transgenic mice and transfected cells (Granic et al., 2010). Aβ cell cycle disruption involved the activation of glycogen synthase kinase 3-beta (GSK-3β), influx of Ca+, and the presence of full length APP as a receptor, positioned to uptake exogenous Aβ. Consequently, Aβ damaged the microtubular system, thereby disrupting motor proteins required by the kinetochores within the spindle apparatus. Once hyper-phosphorylated, tau develops into NFT, one of the hallmarks of AD. Thus, “any dividing precursor or mature cell, in culture or *in vivo*, that cannot undergo proper chromosome segregation will produce defective progeny prone to apoptosis” (Granic et al., 2010).

The role of phosphorylated APP (P-APP) on the cell cycle has been better characterized than that for other APP species. Cyclin Dependent Kinases (i.e.,CDK2, CDK4 and CDK5), increase APP’s phosphorylation at Thr^668^ which increases Aβ production and APP proteolysis by activated caspases during the cell cycle.^76,77,^ Cyclin D-CDK4 and Cyclin E CDK2 are among the Cdks that control G1 progression, and it is generally believed that in AD, neurons are forced out of G0 quiescence to G1 reentry resulting in lethal cell cycles (Park et al., 2000; Majd et al., 2019). Increased cell cycle proteins associate with G1/S in the brains of overproducing APP transgenic mice in the vicinity of plaques where maximum levels of Thr^668^ P-APP were detected (Judge et al., 2011). *In vitro*, Thr^668^ was mitosis specific, P-APP localized to the centrosomes, and Thr^668^ phosphorylation in mitosis correlated with increased processing of APP into Aβ and C-terminal fragments of APP. Further, the expression of Aβ and C-terminal APP fragment was prevented by pharmacological inhibition of G1/S phase (Judge et al., 2011). Phosphorylation of APP, and generation of Aβ and C-terminal fragments localized to G1/S. These findings support APP’s potential contribution to microcephaly early in development: If G1 is arrested, cells go on to G0 and differentiate into glia, causing net neuronal loss (Ohnuma and Harris, 2003). For mature, post mitotic neurons, a return to cell cycle activation would favor apoptosis and early onset neurodegeneration, as seen in T21 and AD (Vincent et al., 1997; Malik et al., 2008; Judge et al., 2011). Also of interest is the localization of P-APP to the centrosome, the site of microcephalic genes (Gilmore and Walsh, 2013).

CDK2, a controller of G1/S transition, also inhibits Thr^668^ phosphorylation of APP, whereas when CDK2 associates with cyclin E, it enables transition of cells through the G1/S cycle (Judge et al., 2011). One can imagine that increase in APP would overwhelm cell cycle mechanisms, favoring early termination of G1 and reduced neurogenesis. Further, localization of P-APP to the centrosome suggests a role for P-APP in microtubule spindle assembly. Of note, APP can be phosphorylated at Thr^668^ not just by cyclin dependent kinases, but by other kinases including GSK-3β, JNK, CDK5 and dual-specificity tyrosine phosphorylation regulated kinase (DYRK1A; Muresan and Muresan, 2007; Wegiel et al., 2011). A feedforward mechanism, wherein APP phosphorylation leads to increased levels of intracellular and extracellular Aβ and induces cell cycle activation with resultant neurodegeneration/cell death has been proposed (Judge et al., 2011). Recent work has linked P-APP to G2/M phase (Zhang et al., 2019). Whether P-APP acts on G1/S or G2/M, the result would be a prolonged wait for cells to enter M, elongating the cell cycle, resulting in a smaller brain. Other mechanisms which would lengthen the cell cycle include initiation of DNA repair for damaged cells which must take place before cell cycle entry. *In vitro* skin biopsied fibroblasts from individuals with T21 revealed an increase in DNA damage and prolongation of the cell cycle duration attributable to DNA repair mechanisms activated to eliminate this damage (Weirich-Schwaiger et al., 1994). These mechanisms could elongate the cell-cycle duration to give repair enzymes enough time to work, perhaps reducing the number of cell cycles.

**Figure 1:**
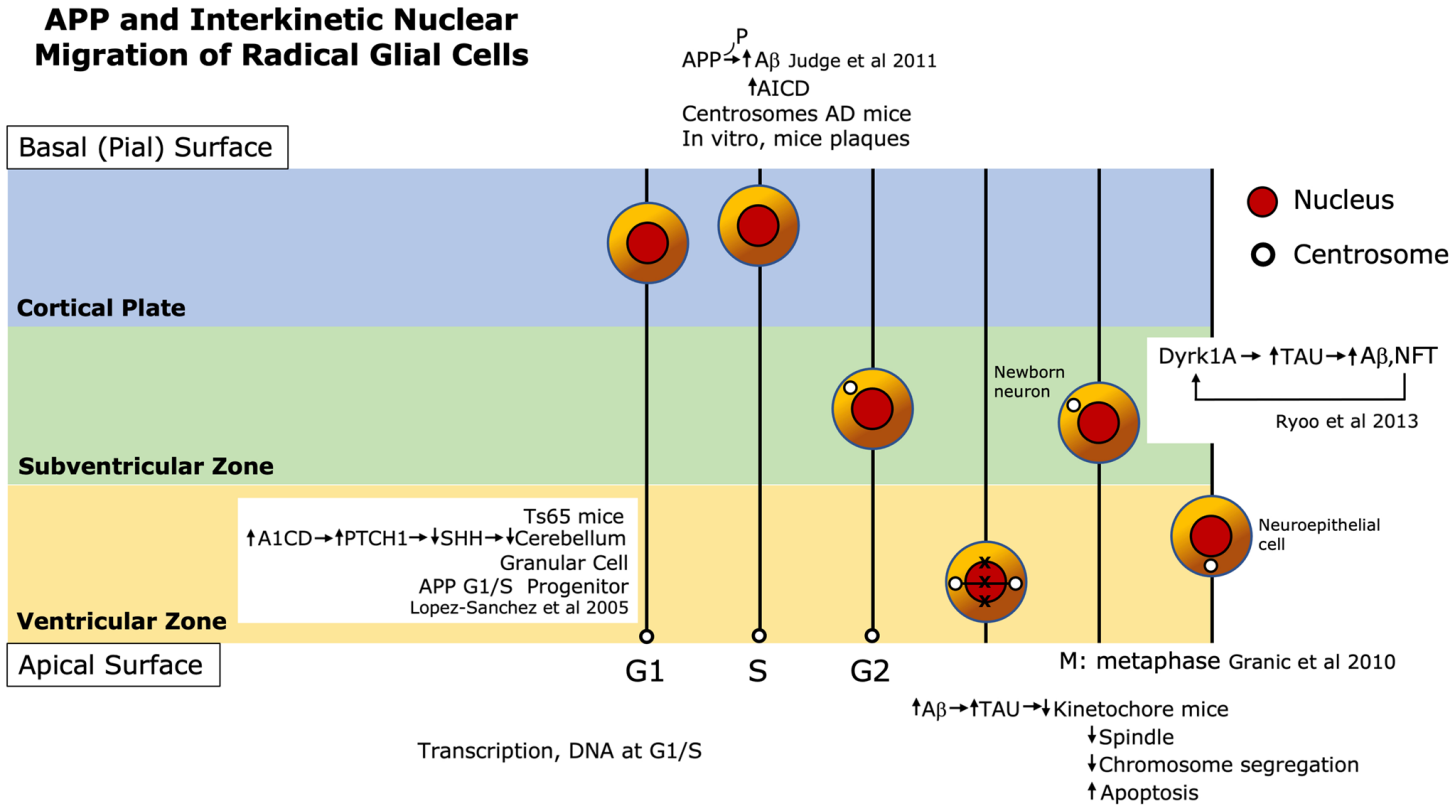
Amyloid-β precursor protein (APP) metabolite Aβ contributes to microcephaly via pathways through neurogenesis and the cell cycle. Judge et al. (2011) showed cell cycle inhibitor phosphorylates APP resulting in a shorter cell cycle, more Aβ, and resultant neurotoxicity and apoptosis, *in vitro* and in AD mice. Lopez-Sanchez et al. (2005) found increased APP intracellular domain (AICD) resulting from increased APP, inhibits Sonic hedgehog (SHH), which causes overexpression of Patched, SHH receptor (Ptch1) contributing to a small cerebellum in T21 mice. Granic et al. (2010) observed that increased Aβ causes disruption of the kinetochore and spindle at the metaphase plate causing chromosome missegregation and ultimately, apoptosis in AD mice. Ryoo et al. (2008) found that DYRK1A increases Tau which increases Aβ that, in turn, may increase DYRK1A in T21 mice.

As overall size of the brain is regulated by cell cycle machinery, different cycle elements could control growth in specific brain areas (Ohnuma and Harris, 2003). Reduction of the size of the cerebellum is seen in T21, and in the Ts65Dn mouse model, and to a lesser extent, the T21 Ts1Cje mouse model. Of interest, APP is represented in the Ts65Dn (three copies), but less in the Ts1Cje (two copies) genotype (Creau, 2012). Sonic hedgehog (SHH) is a growth factor that activates granular cell progenitor production in the cerebellum and this pathway is deficient in Ts65Dn mice. Inhibition of the SHH pathway occurs with overexpression of the AICD fragment of APP (Trazzi et al., 2011). Acting as a transcription regulator, AICD binds to and enacts overexpression of Patched, SHH receptor (Ptch1), a promotor and histone hyperacetylator. The dose effect of AICD on the SHH pathway has been attributed to the smaller size of the Ts65Dn compared to the Ts1Cje cerebellum (Olson et al., 2004), and supports another APP mechanism of reduced brain growth. The effect of APP metabolites on radial glial cells undergoing neurogenesis within the cell cycle is represented in Figure 1.

### Microcephaly in ASD

It has long been thought that individuals with microcephaly and ASD often have an associated known genetic condition. Indeed, known genetic conditions associate with ASD and microcephaly include Rett syndrome, Angelman syndrome, dup 15q11.2-q13 (dup 15), Cornelia de Lange syndrome and T21. Most commonly children with these conditions exhibit acquired rather than congenital microcephaly, i.e., normal birth OFC followed by reduced growth of the brain and head. Genetic source notwithstanding, a surprisingly high percentage of microcephaly recently was reported in a non-syndromic Swedish group of preschool children (*n* = 112) with ICD-10 ASD: 6.7% of the boys and 8% of the girls, whereas only 1.2% of the boys had macrocephaly. None of these children were born microcephalic (Cederlund, 2021).

Genetic causes of congenital microcephaly will first be described to highlight known mechanisms contributing to microcephaly to understand how APP metabolites, particularly Aβ can reduce brain development. Next, T21 and its relationship to ASD, microcephaly, Aβ, tau protein and DYRK1A and will be discussed. Finally, APP metabolites, including Aβ as studied in other genetic conditions associated with ASD with microcephaly will be presented.

### Primary microcephaly

The most common primary (not involved with other syndromes) microcephaly genes are WDR62, ASPM, MCPH1, CDK5RAP2, STIL, CEP152. CENPJ, and CEP63. All involve centrosome abnormalities that impact the mitotic spindle and disrupt mitosis (Gilmore and Walsh, 2013). However, disruption of mitosis alone does not explain why the majority of individuals with primary microcephaly are not small people, i.e., they do not show somatic undergrowth. Other causes, such as mutations in DNA repair pathways leading to programmed cell death (apoptosis), associate with microcephaly (Gilmore and Walsh, 2013), and may be more targeted to brain growth. Different repair mechanisms respond to different type of DNA damage and often involve the cell cycle. For example, a break in double-stranded DNA must be recognized, and the repair mechanism is then activated, which can cause arrest of the cell cycle so that the damage is not propagated via DNA replication or mitosis. This break also provides time for repair. Apoptosis may be activated if the damage is significant. Primary microcephaly has been associated with mutations in the non-homologous end joining (NHEJ)1 gene where double strand DNA breaks are repaired by trimming, then bringing the ends together and ligating the strands. NHEJ knockout mice have significant apoptosis in differentiated neurons around mid-gestation, similar to human microcephaly (Gao et al., 1998). Further, the apoptosis associated with this lack of DNA repair was likely mediated via tumor suppressor p53 (Frank et al., 2000). Upon severe DNA damage, p53 becomes phosphorylated and then activated to induce apoptosis related genes (Frank et al., 2000).

### T21, APP and microcephaly

T21 is the leading genetic cause of intellectual deficiency, with an incidence of 1 in 800 births (Haydar et al., 2000). T21 individuals carry three copies of at least some portion of chromosome 21 in all of their cells due to meiotic chromosome mis-segregation in one of their germ cells, usually obtained from their mother (Granic et al., 2010). The gene encoding APP lies on chromosome 21 and an extra copy contributes to early onset increased levels of Aβ peptides in individuals with T21 (Wegiel et al., 2011). Increased levels of Aβ 42 occur in fetal T21 brain tissue (Teller et al., 1996), and Aβ peptides occur in brains from children ages 8–12 yrs. with T21 (Leverenz and Raskind, 1998). Fifty percent of T21 subjects younger than 30 yrs. show Aβ-positive plaques (Lemere et al., 1996). Individuals with T21 invariably develop AD symptoms and brain pathology by the age of 30–40. APP and Aβ peptides induce increased phosphorylation of microtubule associated protein tau, and the toxicity of Aβ depends upon the presence of tau (Granic et al., 2010). This scenario contributes to AD neuropathology, tau microtubule pathology, and dementia in older individuals with T21.

In T21, a small head is noted at birth: usually −0.5 SD at birth decreasing to − 2.0 SD at 4 years of age (Myrelid et al., 2002). This reduction in brain growth reflects disruption first in neurogenesis, and later in normal processes that result in neurodegeneration (Dowjat et al., 2007). Compared to brains of typically developing individuals, brains of individuals with T21 are both smaller and show specific structural differences (Haydar et al., 2000). Overall, there is delayed and disorganized cortical lamination and decreased number of neurons in the cortex. The dentate gyrus of the hippocampus is smaller and hypocellular, and the cerebellum is smaller and hypomorphic. Hypotonia, seen in the majority of individuals with T21, has been attributed to the decreased size of the cerebellum (Lott, 2012). The reduction in the number of neurons and the smaller brain structures can be detected during fetal development suggesting the stronger contribution from a neurogenesis defect over neurodegeneration (Contestabile et al., 2007).

### T21 and ASD

Not long ago it was assumed that individuals with T21 were “protected against impairment of social interaction and ASD” (Moss, 2017). However, prevalence rates of ASD in individuals with T21 range from 16 to 42% (Richards et al., 2015; Oxelgren et al., 2017), which is a much higher risk compared to the general population (1%; Baird et al., 2006). Use of diagnostic or clinical cut off scores or normative base groups that do not altogether account for the baseline skills possessed by those with T21 may account for this sizable prevalence variability (Moss, 2017). For example, children with T21 *without* ASD showed good social awareness and motivation but lacked social cognition that allows accurate interpretation of verbal and nonverbal skills such as required to understand humor, sarcasm or a partner’s intentions (Channell et al., 2015). Children with T21+ ASD are more likely to have severe intellectual deficiency, express limited shared affect and lack functional communication that leads to maladaptive behavior (Rachubinski et al., 2017; Godfrey et al., 2019). There are very few reports about the molecular underpinnings of T21 + ASD. The general 50% increase in trisomic gene expression on Chromosome 21, the home of other candidate ASD genes such as WDR4, CBS, and PTTG1IP, may contribute to T21 + ASD (Rachubinski et al., 2017). APP and DYRK1A are among those genes with increased expression, and have a synergistic relationship in promoting Aβ and tau phosphorylation.

Further, these growth proliferation defects have been hypothesized as one of the possible explanations for the surprising finding of fewer solid brain tumors such as medulloblastoma and neuroblastoma found in individuals with T21 (Creau, 2012).

### Apoptosis determinants of microcephaly in T21

APP regulates apoptosis in the neuron (Neve et al., 2001), and oligomerized and/or polymerized Aβ peptides are toxic to neurons (Granic et al., 2010). Human neurons from 16 to 19 week T21 fetuses, containing Aβ peptides appeared to undergo apoptosis, attributable to a defect in management of reactive oxygen species (ROS; Busciglio and Yankner, 1995). Aβ peptides also inhibited neurogenesis and decreased proliferation in human neurosphere culture and killed mature neurons (Haughey et al., 2002). Aβ peptide-mediated destruction was due to disruption in calcium homeostasis, specifically by inducing membrane lipid peroxidation which in turn resulted in impairment of membrane ion and glucose transport causing increased intracellular Ca^2+^ and apoptosis. Newly generated WT mouse embryo neurons and NPC are killed by Aβ peptides and it was reported that NPC died via a caspase dependent pathway (Millet et al., 2005). Aβ peptides trigger caspase and non-caspase pathways leading to apoptosis (Head et al., 2002).

Accumulation of intracellular Aβ peptides occurs at an early age in T21 (infants and children), compared to the general population (Head et al., 2002, 2018). Intracellular Aβ, observed prior to extracellular Aβ accumulation, may be important in the developmental course of T21. Aβ accumulation appears to be caspase dependent and associated with neuronal loss (Head et al., 2002). In T21, Aβ42 diffuse plaques occur before fibrillar senile plaques containing dysfunctional neurites and NFT. In AD, Aβ42 plaques are seen more frequently than Aβ40 plaques at all ages. Further, in AD, there is altered neuronal protein phosphorylation with elevation of GSK-3β. which phosphorylates tau, disrupting microtube-based motor protein axonal transport, causing synaptic problems and ultimately cell death (Brady and Morfini, 2017). This interplay advances discussion of the effect of the regulation of tau on brain growth in T21.

### Tau protein, APP, and DYRK1A

Tau protein controls microtubule function including microtubule motor transport, stability and modifications, and regulating the spacing of microtubules within axons (Arbones et al., 2019). Tau isoforms with four (4R) or three (3R) microtubule-binding repeats have been the most studied, with increased 3R tau levels associated with the progression of AD. Aβ peptides, mutant preseniln-1 (a component of δ-secretase), and APP induce increased phosphorylation of the tau present in neurofibrillary tangles of AD (Granic et al., 2010). Specifically, Aβ peptides directly damage the microtubule system by disconnecting tau, facilitating phosphorylation and microtubule destabilization. By way of tau phosphorylation, APP and derivatives induce chromosome mis-segregation that leads to cell cycle disruption, producing defective progeny that is prone to apoptosis (Granic et al., 2010).

Even with evidence showing APP derivatives induce phosphorylation of tau, having three copies of the APP gene is necessary but not sufficient for T21-affected individuals to develop AD pathology (Ryoo et al., 2008). Increased APP alone does not cause the AD-like endosome pathology seen in T21 individuals and in the Ts65Dn mouse, implying that one or more additional genes on chromosome 21 must contribute to AD pathology in T21 (Ryoo et al., 2008). The gene encoding DYRK1A, located within the T21 critical region (comprising the most deleterious triplicated genes), provides a functional link between T21 and AD (Ryoo et al., 2008). It is believed that DYRK1A may “play a significant role in developmental brain defects, and in the early onset of neurodegeneration neuronal loss and dementia in T21” (Wegiel et al., 2011). DYRK1A accomplishes this by inducing hyper-phosphorylation of tau and APP, leading to increased NFT and Aβ peptides, respectively.

### DYRK1A in T21

In humans, DYRK1 is found in astrocytes, ependymal, and endothelial cells; it has numerous substrates and contributes to many cellular pathways. DYRK1A is a dual kinase that phosphorylates its own activation loop via a tyrosine residue and phosphorylates its substrates on threonine and serine residues (Laham et al., 2021). The DYRK1A RNA expression profile shows a strong presence in early brain development in murine and chick models, before the onset of neurogenesis, and in neuronal progenitors and differentiated neurons and peaks around birth (Arbones et al., 2019). DYRK1A substrates include chromatin regulators and transcription factors suggesting that DYRK1A is a regulator of gene expression. DYRK1A phosphorylates notch transcription factor and reduces notch signaling in neural cells which causes cell differentiation. It is a negative regular of cell cycle progression, repressing regulators of G1/S phase, inducing G0/G1 arrest (Arbones et al., 2019).

DYRK1A is a dose sensitive enzyme. Both low and high expression cause different effects (Laham et al., 2021). DYRK1A gene truncation and a heterozygous DYRK1A genotype—known as DYRK1A *haploinsufficiency* syndrome, is associated with microcephaly, speech delay, dysmorphia, and autistic features (Ji et al., 2015). In contrast, DYRK1A is 1.4-fold *overexpressed* in T21 lymphoblastic cells as a result of the three copies of the *DYRK1A* gene (Wegiel et al., 2008). Significantly, some attribute the cognitive dysfunction seen in T21 to DYRK1A overexpression (Laham et al., 2021).

In human T21 and mouse brain, most DYRK1A is in filamentous actin, neurofilaments, and tubulin in the cytoskeleton with the remaining present in the cytosol and nucleus (Wegiel et al., 2011). Overexpression of DYRK1A in T21 contributes to cell-type, cell compartment, and region specific changes that impact early brain development and susceptibility to early neurodegeneration (Wegiel et al., 2011). In murine models, DYRK1A overexpression instills inhibition of proliferation with premature differentiation, and lengthening of G1/S with early cell cycle exit likely due to reduction of Cyclin D1 (Najas et al., 2015). This resulted in reduced production of neurons. A dose dependent effect for DYRK1A protein exists. Greater gene doses (as occurs in T21) have more of an effect than moderate doses. Interestingly, genetic normalization of the DYRK1A gene dosage in Ts65DN embryos increased the amount of Cyclin D1 to normal and returned production of early-born cortical neurons to normal (Najas et al., 2015). This shows the early effect of DYRK1A on T21 neuronal cell proliferation and differentiation, and how DYRK1A may contribute to a reduced number of neurons produced in early brain development. Further, this demonstrates that DYRK1A reduction is potentially therapeutic. Finally, DYRK1A contributes to continued neuronal loss during early onset neurodegeneration leading to AD in T21 (Wegiel et al., 2011; Najas et al., 2015).

DYRK1A may be a primary risk factor in causing the enhancement of both β-amyloidosis and NFT and “DYRK1A and Aβ peptides may positively feedback and accelerate Aβ production” (Wegiel et al., 2011). Specifically, DYRK1A phosphorylates APP at Thr^668^ which facilitates BACE1 and y-secretase cleavage, resulting in increased production of Aβ peptides (Ryoo et al., 2008). Stimulation of DYRK1A mRNA by Aβ peptides in neuroblastoma cells demonstrate that Aβ increases expression of DYRK1A. In this way, elevated Aβ peptides result in increased expression of DYRK1A gene and hyperphosphorylation of tau. Inhibition of DYRK1A can reduce Aβ accumulation and tau phosphorylation in mice (Branca et al., 2017). Finally, DYRK1A is overexpressed in individuals with T21 and AD compared to AD by several-fold increase, measured as the number of DYRK1A-positive NFTs in human T21/AD brain compared to human AD brain tissue samples (Wegiel et al., 2008). If DYRK1A is a significant risk factor for AD, extra DYRK1A expression in T21/AD even over AD without T21 could explain or at least contribute to the much earlier onset of pathology and dementia in T21 individuals.

## Amyloid-β precursor protein non amyloidogenic processing pathways can also contribute to microcephaly in various neurodevelopmental disorders

### 15q11-q13 duplication syndrome

Maternal duplication of 15q11-q13 is a neurodevelopmental disorder, affecting the supernumerary isodicentric chromosome 15 Prader-Willi/Angelman syndrome (idic; Ebert and Greenberg, 2013) critical region. Clinical features include intellectual deficiency, seizures, autistic features, hypotonia, and hyperactivity. Microcephaly can associate with this disorder, but it is not clear if this is acquired. A higher rate of microcephaly compared to macrocephaly (16.8% compared to 2.8%) was found in a study of 107 dup 15q11-q13 cases, but the lowest age was 5 years, so it was not clear if this was congenital or acquired (Schroer et al., 1998). A sample of individuals with dup 15q11.2-q13 exhibited microcephaly in the majority of cases (Wegiel et al., 2012; Frackowiak et al., 2020). However, the youngest patient in their series was 9 yrs., so it is uncertain if microcephaly was acquired. A 9 months-old-boy with dup 15q11-q13 and microcephaly presented with delayed motor development, but had no abnormal clinical signs reported at birth, with a normal birth weight, and a normal brain MRI at 4 months of age (Park et al., 2013). This boy’s microcephaly appears to have been acquired by 9 months of age. The ASD phenotype associated with dup 15q11-q13 has been attributed to dysfunction in transcription (Urraca et al., 2013), but the occurrence of acquired microcephaly, as seen in other neurodevelopmental disorders of transcription, awaits confirmation.

Wegiel et al. (2012) and Frackowiak et al. (2013, 2020) studied brain specimens via confocal microscopy in 9 children and adults (ages 9–39) with dup 15q11.2-q13. Many of these subjects had early onset, intractable epilepsy. The confocal microscopy revealed enhancement of intraneuronal N-terminal APP, i.e., the P3 fragment. They found the accumulation of P3 in nearly all cortical and subcortical structures, with lessor but similar results for an idiopathic autistic cohort, compared to controls (Wegiel et al., 2012; Frackowiak et al., 2013, 2020). These results were consistent with the increased level of the plasma sAPP a which we found in ASD (Sokol et al., 2006; Ray et al., 2016). P3 is the product of sequential cleavage of APP by α-secretase and γ-secretase; it is not produced by β-secretase. It is distinct from Aβ in activity and structure, and it is not associated with AD or neurodegenerative disorders. The P3 fragment was found in neuronal endosomes, autophage vacuoles, lysosomes and lipofusion in dup 15q11.2-q13 and autistic samples. In a subsequent study, oxidative stress, in the form of oxidatively modified lipids, were associated with the increase in P3, and the resultant modified lipids, but not P3, were detected in all mitochondria (Frackowiak et al., 2013). This is consistent with the finding from a large meta-analysis that APP products, including Aβ are too large to invade the mitochondria membrane, but causes damage indirectly (Kaminsky et al., 2015).

Alternately, the expression of the P3 component may have been in response to stress. It was hypothesized that this product of the nonamyloidogenic pathway, was a neuroprotective response to the refractory epilepsy and head banging in this severely affected cohort. Indeed, Sudden Unexpected Death in Epilepsy (SUDEP) was the cause of death in 6 out of 9 of the dup 15q11.2-q13 patients sampled. High levels of P3 peptide reactivity appeared in the subjects’ prefrontal cortex GABAergic neurons (Frackowiak et al., 2013). Group comparison frequency/intensity of neurons with above average P3 immunoreaction was 5.1% for control, 9.3% for idiopathic ASD, and 25.5% for dup15q11.2-q13. P3 in childhood ASD may enhance formation of ROS, which results in further formation of P3 peptides in a vicious cycle, contributing to neuron dysfunction, which appears accentuated in dup15q11.2-q13 (Wegiel et al., 2012; Frackowiak et al., 2020). The finding of increased P3 reactivity in dup15q11.2-q13 supports the association between P3 and microcephaly, potentially as an additional pathway to that of associating Aβ and microcephaly. However, this contradicts the many other associations between the anabolic APP processing path, which produces sAPPα, the associated P3, and a tendency far more toward macrocephaly than microcephaly. It is unlikely that a single (anabolic) pathway can alternatively lead to microcephaly *and* macrocephaly.

### Angelman’s syndrome

Seventy-five percent of AS cases occur when there is a microdeletion of region 15q11-13 of the maternally-derived chromosome 15 (Wink et al., 2015). Chief clinical symptoms are developmental delay without acquisition of speech, acquired microcephaly, autistic features, ataxia, hypotonia, frequent smiling, unprovoked laughter and seizures. Born normocephalic, they acquire microcephaly usually by 2 years of age (Kishino et al., 1997).

Abnormality in the 15q11-13 AS region includes deletion of the maternally derived UBE3A gene. Since the maternal UBE3A gene and not the paternal gene, allows transcription of the protein, i.e., imprinting, the neuron does not make the protein. UBE3A has been shown to interact with primary microcephaly protein ASPM which localizes to centrosomes, regulating segregation (Singhmar and Kumar, 2011). Further, UBE3A knockdown led to disruption of mitosis causing chromosome mis-segregation, abnormal cytokinesis, and apoptosis (Singhmar and Kumar, 2011). Loss of UBE3A function may lead to defective chromosome segregation that would generate inviable aneuploid cells. The resultant increased cell death and reduced neural progenitor cells would then lead to microcephaly. Such cell cycle processes occur over time, which may account for the acquired rather than primary microcephaly seen in AS (Singhmar and Kumar, 2011).

The UBE3A protein acts in a process called ubiquitination that “tags” other proteins for degrading and recycling. Problems with ubiquitination causes proteins to accumulate that can hurt neuron function. Neddylation is a major regulatory pathway for ubiquitination. Ubiquitination dysfunction can play a role in neurodegenerative diseases (Chen et al., 2012). Specifically, APP appears to play a key role in the APP-binding protein 1 (APPBp1) activated neddylation pathway that promotes cell cycle progression through the S/M checkpoint, which may regulate stem cell renewal (Ryoo et al., 2008). Further, APPBp1 joins with UBE3 to form an enzyme that activates nedd8, causing neddylation. Therefore, dysfunction of neddylation is involved in both AD and AS. UBE3A was reduced in an AD mouse model Tg2576, which overexpresses human APP_695_, amassing Aβ peptide in brain (Olabarria et al., 2019). Can decreased UBE3A, as seen in AS, contribute to increased Aβ peptide in this disorder? Plasma levels of APP total, sAPP a , Aβ 40 and Aβ 42 were elevated in a group of individuals with AS, compared to age matched controls (age 3–29 years; Erickson et al., 2016). It appears that the N-terminal APP is at large in AS, perhaps as neuroprotection against the deleterious Aβ peptides also increased in this group. P3, however, was not measured. Further study into the effect of Aβ peptide on AS microcephaly is warranted, particularly as defective neddylation may lead to increased Aβ (Olabarria et al., 2019).

### Rett syndrome

A progressive neurodevelopmental disorder, Rett syndrome mostly affects females. Most cases are caused by mutation of the ethyl-CpG binding protein 2 (MeCP2) gene on the X chromosome. Because of its variable course and severity, Rett syndrome most likely represents a spectrum of disease as the MeCP2 gene mutation involves random X-inactivation that can cause different phenotypes (Moss and Howlin, 2009). Affected infants generally show normal development until toddler age, when head growth slows down at 3 months of age and hypotonia can be seen before 6 months (Moss and Howlin, 2009). Social regression, reduced eye contact and delayed motor function follows, with the distinctive feature of loss of purposeful hand movements. Altogether, children with Rett syndrome are afflicted with seizures, breathing irregularities, growth disorder and intellectual deficiency (Petriti et al., 2023). Loss of function MeCP2 associates with girls and gain of function MeCP2 associates with boys; both show acquired microcephaly and autistic features.

MeCP2 is a protein that binds to methylated CpG nucleotide sequence to control transcription via epigenetic modification (Kim et al., 2019). MeCP2 regulates histone deacetylase-dependent histone deacetylation. It bridges histone and DNA methylation, and activates CREB1-dependent transcription (Kim et al., 2019). Acquired microcephaly in Rett Syndrome is associated with small neuronal body size and denser packing of cells throughout the brain (Armstrong, 1992), but the causal path to microcephaly due to MeCP2 deficiency is unknown. In cultured neurons and neuronal tissue, MeCP2 overexpression increased apoptotic cell death (Petazzi et al., 2014). Further, MeCP2 represses the α-secretase ADAM10 in human and mouse neural progenitor cells (Wang et al., 2019). Reduction of ADAM10 would favor Aβ production. Additionally, MeCP2 expression and phosphorylation levels were increased in CA1 hippocampus of A β injected rats (Zikopoulos and Barbas, 2013). Total and phosphorylated MeCP2 levels were increased in human AD brain tissue and in the hippocampus of transgenic mouse overexpressing the human tau gene (Maphis et al., 2017), suggesting that MeCP2 may regulate tau pathology. An increase in MeCP2-e2 isoform was shown to mediate A β -induced apoptosis in cultured cortical neurons, suggesting a potential link between MeCP2 and Aβ (Montgomery et al., 2018). A further link between Rett Syndrome and AD was advanced via observation that microglia appears dysfunctional in both conditions. That is, microglia does not function in Rett’s syndrome to clear cells associated with (normal) apoptosis during early brain growth, nor do they function in AD to clear Aβ (Derecki et al., 2014). However, this science is evolving, and more research is warranted to understand the link between Aβ and Rett syndrome.

### Caveat: Is APP a cause or effect of epilepsy in microcephaly?

APP abnormality, per say, may be causal as significant mutations in the APP gene have been associated with microcephaly. For example, homozygous nonsense mutations in APP found in a child was associated with microcephaly, decreased somatic growth, hypotonia, developmental delay, thinning of the corpus callosum, and seizures (Klein et al., 2016). In another child, a nonsense mutation in Ox-2 antigen domain of APP was associated with microcephaly, seizures, bilateral cataracts truncal hypotonia with spastic quadriparesis (Nguyen et al., 2017).

Epilepsy is another comorbidity, besides microcephaly, which accompanies T21, dup15q11.2-q13, Angelman’s and Rett’s syndrome. Indeed, SUDEP is associated with dup 15q11.2-q13 and was the cause of death for several of the children with dup 15q11.2-q13 in the studies cited above (Wegiel et al., 2012; Frackowiak et al., 2013). High levels of phosphorylated APP and β-secretase have been found in temporal lobe samples excised from individuals with medically refractory temporal lobe epilepsy (Gourmaud et al., 2020). It may be possible that any link between Aβ40/Aβ42 and these genetic conditions in fact are due to alterations in APP processing as a result of intractable seizures.

### Summary: APP in syndromic ASD microcephaly

As demonstrated by increased APP expression in the T21 prototype, resulting in excessive APP metabolites, especially Aβ peptides, may contribute to microcephaly via disruption in neurogenesis, elongated G1/S cycle, arrested cell cycle, promoting apoptosis (Figure 1). Specific mechanisms may be in play that cause reduction in the size of brain regions such as the effect of excessive AICD that activates Ptch1, which in turn suppresses SHH, contributing to hypocellularity seen in the T21 cerebellum. Processes linked to later neurodegeneration in T21 involve DYRK1A phosphorylation of tau that results in further accumulation of NFT and Aβ. The exact mechanism that APP and derivatives additionally contribute to autistic features seen in conditions of microcephaly remains to be discovered. Epilepsy, associated with several genetic conditions, may also contribute to alterations in APP processing. APP and Aβ are only beginning to be studied in neurodevelopmental disorders of acquired microcephaly such AS and dup15q11-q13 where P3 has been detected as a potentially neuroprotective response to accumulation of Aβ peptide. The effect of APP in Rett disorder is less well characterized, but MeCP2 may be a regulator of tau. Future work could focus on specific routes APP may take to directly affect centrosomes by way of CDKRAP2, which has been associated with microcephaly, such as the astral microtubular pathway that directs proteins from the nuclear membrane to the centrioles that comprise the centrosome. Uncovering APP’s role in syndromic ASD may lead to identification of common pathways that impact nonsyndromic ASD with microcephaly.

Beyond genetics, intersectionality between ASD and AD can emerge at the protein translation and translocation level. For example, we have recently suggested a potential novel avenue of scientific research, whereby APP translation is multiply TrAPPed (Translation of APP elevation and decline) and influences the etiology of ASD and AD. In short, the fates of ASD and AD are trapped in mis-regulation of APP mRNA transport, translation, and processing (Lahiri et al., 2021). Likewise, in addition to genetics, epigenetics plays a critical role. Environmental factors, particularly gene–environment interaction, beginning in early childhood may influence neurological disorders. In the future, this intersectionality between early-life, environment, ASD, and AD warrants further research (Lahiri et al., 2022).

## Author contributions

DS: original draft preparation. DL: critical revision of manuscript. All authors contributed to the article and approved the submitted version.

## Funding

This work was supported by NIA/NIH grants related to AD research (P30AG010133, P30AG072976, R01AG051086-06, R56AG072810, 2R56AG051086-06A1, and R21AG074539).

## Conflict of interest

The authors declare that the research was conducted in the absence of any commercial or financial relationships that could be construed as a potential conflict of interest.

## Publisher’s note

All claims expressed in this article are solely those of the authors and do not necessarily represent those of their affiliated organizations, or those of the publisher, the editors and the reviewers. Any product that may be evaluated in this article, or claim that may be made by its manufacturer, is not guaranteed or endorsed by the publisher.
